# Mesenchymal Stromal Cell-Derived Extracellular Vesicles Reduce Neuroinflammation, Promote Neural Cell Proliferation and Improve Oligodendrocyte Maturation in Neonatal Hypoxic-Ischemic Brain Injury

**DOI:** 10.3389/fncel.2020.601176

**Published:** 2020-12-10

**Authors:** Nicole Kaminski, Christian Köster, Yanis Mouloud, Verena Börger, Ursula Felderhoff-Müser, Ivo Bendix, Bernd Giebel, Josephine Herz

**Affiliations:** ^1^Department of Pediatrics I, Neonatology and Experimental Perinatal Neurosciences, University Hospital Essen, University Duisburg-Essen, Essen, Germany; ^2^Institute for Transfusion Medicine, University Hospital Essen, University Duisburg-Essen, Essen, Germany

**Keywords:** mesenchymal stem/stromal cells (MSC), extracellular vesicles (EV), MSC-EV, neonatal hypoxia-ischemia, oligodendrocyte maturation, neuroregeneration, neuroinflammation

## Abstract

**Background:** Neonatal encephalopathy caused by hypoxia-ischemia (HI) is a major cause of childhood mortality and disability. Stem cell-based regenerative therapies seem promising to prevent long-term neurological deficits. Our previous work in neonatal HI revealed an unexpected interaction between mesenchymal stem/stromal cells (MSCs) and the brains' microenvironment leading to an altered therapeutic efficiency. MSCs are supposed to mediate most of their therapeutic effects in a paracrine mode via extracellular vesicles (EVs), which might be an alternative to cell therapy. In the present study, we investigated the impact of MSC-EVs on neonatal HI-induced brain injury.

**Methods:** Nine-day-old C57BL/6 mice were exposed to HI through ligation of the right common carotid artery followed by 1 h hypoxia (10% oxygen). MSC-EVs were injected intraperitoneally 1, 3, and 5 days after HI. One week after HI, brain injury was evaluated by regional neuropathological scoring, atrophy measurements and immunohistochemistry to assess effects on neuronal, oligodendrocyte and vessel densities, proliferation, oligodendrocyte maturation, myelination, astro-, and microglia activation. Immunohistochemistry analyses were complemented by mRNA expression analyses for a broad set of M1/M2- and A1/A2-associated molecules and neural growth factors.

**Results:** While total neuropathological scores and tissue atrophy were not changed, MSC-EVs significantly protected from HI-induced striatal tissue loss and decreased micro- and astroglia activation. MSC-EVs lead to a significant downregulation of the pro-inflammatory cytokine TNFa, accompanied by a significant upregulation of the M2 marker YM-1 and the anti-inflammatory cytokine TGFb. MSC-EVs significantly decreased astrocytic expression of the A1 marker C3, concomitant with an increased expression of neural growth factors (i.e., BDNF, VEGF, and EGF). These alterations were associated with an increased neuronal and vessel density, coinciding with a significant increase of proliferating cells in the neurogenic sub-ventricular zone juxtaposed to the striatum. MSC-EV-mediated neuroprotection went along with a significant improvement of oligodendrocyte maturation and myelination.

**Conclusion:** The present study demonstrates that MSC-EVs mediate anti-inflammatory effects, promote regenerative responses and improve key developmental processes in the injured neonatal brain. The present results suggest different cellular target mechanisms of MSC-EVs, preventing secondary HI-induced brain injury. MSC-EV treatment may be a promising alternative to risk-associated cell therapies in neonatal brain injury.

## Introduction

Neonatal brain injury caused by hypoxia-ischemia (HI) is a leading cause of childhood mortality and neurodevelopmental morbidity, associated with cerebral palsy, epilepsy, and visual impairment as well as cognitive and motor deficits in later life (Ahearne et al., 2016). To date, the only clinically recommended and established therapeutic intervention is a hypothermia (HT) treatment initiated within the first 6 h of life. However, 40–50% of cooled infants still suffer from major neurological problems (Azzopardi et al., 2014). Thus, new and/or additional treatment strategies aiming at the attenuation of injury and the enhancement of repair mechanisms are urgently required.

Taking into account that acute neuroprotective treatment options like HT are significantly limited, regenerative therapies to promote endogenous repair by the use of mesenchymal stem/stromal cells (MSC) have gained major interest in the past. Transplantation of bone marrow-derived MSCs in a variety of animal models, including murine models of adult and neonatal ischemic brain injury has been shown to improve neurobehavioral deficits involving anti-apoptotic, pro-regenerative and immunomodulatory effects (Yasuhara et al., 2008; van Velthoven et al., 2010; Jellema et al., 2013; Doeppner et al., 2015; Herz et al., 2018b). However, MSCs still harbor unforeseen risks such as modulation of MSC function by an altered *in vivo* microenvironment caused by HT (Herz et al., 2018b). Therefore, new concepts circumventing potential risks associated with the cells' plasticity after release into complex *in vivo* systems are needed. Increasing evidence suggests that therapeutically relevant effects are mediated by MSC-derived extracellular vesicles (MSC-EVs) (Lener et al., 2015; Borger et al., 2017; Witwer et al., 2019). Containing lipids, proteins and RNA, MSC-EVs participate in complex intercellular communication processes in a variety of physiological and pathophysiological processes (Ludwig and Giebel, 2012; Lener et al., 2015; Borger et al., 2017; Witwer et al., 2019). MSC-EVs provide important advantages over cellular therapeutics, because they are not self-replicating, can be well-characterized *in vitro* and can be sterilized by filtration. Furthermore, unlike cells, EVs hardly sense environmental conditions and therefore do not change their function in a context-dependent manner (Lener et al., 2015).

Neuroprotective, neuroregenerative, and immunomodulatory effects of MSC-EVs have been demonstrated in different models of adult and neonatal brain injury (Doeppner et al., 2015; Drommelschmidt et al., 2016; Ophelders et al., 2016; Gussenhoven et al., 2019; Sisa et al., 2019; Wang et al., 2020). In a model of adult brain ischemia, we demonstrated that MSC-EVs improve neuro-angiogenesis and associated functional recovery, which was accompanied by immunomodulatory effects in the periphery and the brain (Doeppner et al., 2015; Wang et al., 2020). To evaluate the therapeutic potential of MSC-EVs in perinatal brain injury we further analyzed effects of systemically administered MSC-EVs in a model of inflammation-induced preterm brain injury revealing anti-apoptotic effects, enhanced myelination, and reduced astro- and microgliosis, resulting in improved cognitive function in later life (Drommelschmidt et al., 2016). Even though our previous studies (Doeppner et al., 2015; Drommelschmidt et al., 2016) hold great promise for a successful outcome in neonatal HI-induced brain injury, EVs' target mechanisms most probably depend on the underlying pathology. Due to different developmental stages and different pathologies, findings in the previously investigated models cannot be extrapolated unequivocally. For instance, in a fetal ovine model of HI, MSC-EV administration did not significantly modulate microglia activation and myelination in spite of improved functional outcome (Ophelders et al., 2016). In contrast to this report, a recent study in a rodent term-equivalent model of HI demonstrated reduced microglia activation after intranasal MSC-EV administration (Sisa et al., 2019). Common to both studies is the focus on short-term outcome. The impact on sub-acute inflammatory responses, regenerative processes, initiated in the delayed disease phase, and potential modulation of developmental processes like oligodendrocyte proliferation and differentiation remain unknown.

In the present study, we investigated effects of systemically administered MSC-EVs on delayed structural brain tissue injury and cellular responses for a broad variety of cells including neurons, oligodendrocytes, endothelial cells, astrocytes, and microglia to get deeper insight into MSC-EVs' target mechanisms in neonatal HI-induced brain injury.

## Materials and Methods

### Animal Care and Allocation

Experiments were performed in accordance to the Animal Research Reporting of *In Vivo* Experiments (ARRIVE) guidelines with government approval by the State Agency for Nature, Environment and Consumer Protection North Rhine-Westphalia. C57BL/6J mice were bred in house and kept under a 12 h light/dark cycle with food and water *ad libitum*. Bodyweight of pups was recorded at postnatal day 9 (P9), P10, P11, and P12. A total of 43 C57BL/6 mice (*n* = 20 female and *n* = 23 male) derived from 6 litters were enrolled. A pilot study with a small cohort of animals [1 litter, *n* = 7, 3 sham (2 female, 1 male), 4 HI (1 female, 3 male)] was performed to characterize the general impact of HI on major cellular and molecular mechanisms, investigated the present study (Supplementary Figure 1). Data from analysis of MSC-EV treatment effects in HI-injured animals were derived from two independent experiments (with 2 and 3 litters, respectively). For all analyses, animals per litter and experiment were randomly assigned to 3 experimental groups [vehicle *n* = 14, platelet-derived EVs (PL-EV) *n* = 8, MSC-EV *n* = 14] prior to intervention. To control the potential influence of weight and sex, a stratified randomization was performed followed by simple randomization within each block to assign pups to individual groups. Individuals involved in data analysis knew the animals' designation but were blinded to group assignment. In total, 5 animals [13.3%, 2 female (1 vehicle, 1 PL-EV) and 3 male (2 MSC-EV, 1 vehicle)] died. No group differences were observed for weight gain.

### MSC-EV Production and Characterization

MSC-EVs were prepared and characterized as described previously (Kordelas et al., 2014; Doeppner et al., 2015). Briefly, MSCs were raised from human bone marrow aspirates from healthy donors following informed consent according to the Declaration of Helsinki and as approved by the local ethics commission (12-5295-BO). MSCs were propagated in low glucose Dulbecco's Modified Eagle Medium (DMEM) (PAN Biotech), supplemented with self-produced 10% human platelet lysate (PL), 100 U/ ml penicillin-streptomycin-glutamine (Thermo Fisher Scientific, Germany) and 5 IU/ ml Heparin (Ratiopharm, Germany) at 37°C in a 5% CO_2_ atmosphere. MSC-conditioned media (MSC-CM) were harvested from cultures with 50 and 90% confluence, every 48 h. After harvesting, MSC-CMs were spun at 2,000 × g for 15 min (Avanti centrifuge and JS-5.3 rotor; Beckman Coulter). Supernatants were stored at −20°C until further processing. After thawing, debris and larger EVs were removed by 45 min centrifugation at 6,800 × g (Avanti J-26 XP centrifuge using the swing-out rotor JS-5.3; Beckman Coulter) and subsequent filtration of the supernatant through 0.22 μm Nalgene filters (Thermo Fisher Scientific). EVs were enriched by 10% polyethylene glycol 6000 (PEG) precipitation in 75 mM sodium chloride (NaCl). Following over-night incubation at 4°C, samples were spun at 1,500 × g and 4°C for 30 min (Avanti J-26 XP centrifuge with the JS-5.3 rotor) (Ludwig et al., 2018). EV containing pellets were solved in 0.9% NaCl (Braun, Melsungen, Germany). The content of co-precipitated molecules of PEG were reduced by a washing step with 0.9% NaCl and subsequent ultracentrifugation (XPN-80 ultracentrifuge using the tight angle rotor Ti45, Beckmann Coulter; 110,000 × g for 130 min, k-factor: 133). Obtained pellets were solved in NaCl-HEPES buffer (Thermo Fisher Scientific) to a concentration of 4 x 10^7^ MSC equivalents per ml. Fresh PL supplemented media was processed exactly the same to obtain PL-EV control samples. Prepared samples were stored at −80°C until usage. All obtained EV samples were characterized according to the Minimal Information for Studies of Extracellular Vesicles (MISEV) 2018 criteria (Thery et al., 2018), i.e., NTA analyses, western blot and transmission electron microscopy. Using the same MSC-EV samples (line 41.5) as in our previous studies (Doeppner et al., 2015; Drommelschmidt et al., 2016; Wang et al., 2020), we like to refer to Wang et al. for the results of all quality control analyses (Wang et al., 2020).

### Neonatal Hypoxia-Ischemia and MSC-EV Treatment

Hypoxic-ischemic (HI) brain injury was induced in 9-day-old animals as previously described (Reinboth et al., 2016; Herz et al., 2018a,b). Briefly, the right common carotid artery was occluded through cauterization (high temperature cauter, 1,200°C, Bovie, USA) under isoflurane anesthesia (1.5–4 Vol%, total duration of surgery: 5–7 min) followed by 1 h hypoxia (10% O_2_) in an air-tight oxygen chamber (OxyCycler, Biospherix, USA) after 1 h recovery with their dams. Animals were placed on a warming mat (Harvard Apparatus, USA) to maintain nesting temperature during hypoxia (Reinboth et al., 2016). Sham animals were subjected to anesthesia and neck incision only. Perioperative analgesia was ensured by subcutaneous administration of 0.1 mg/kg Buprenorphine. According to our previous study, MSC-EVs (1 × 10^5^ cell equivalents/g bodyweight) or the corresponding amount of PL-EVs were administered intraperitoneally in 10 μl/g body weight 24, 72, and 120 h post-HI (Doeppner et al., 2015). Vehicle treated control animals received the same volume of 0.9% NaCl at the same time points.

### Tissue Preparation, Histology, and Immunohistochemistry

Seven days after HI, mice were deeply anesthetized with chloral-hydrate and transcardially perfused with ice-cold PBS. Brains were removed and snap frozen on dry ice. Tissue injury was assessed and scored on cresyl violet stained 20 μm cryostat sections as previously described (Sheldon et al., 1998; Reinboth et al., 2016). Briefly, 9 regions were scored: the anterior, middle and posterior cortex, CA1, CA2, CA3 and dentate gyrus of the hippocampus and the striatum. Each region was given a rating from 0 to 3 (0—no detectable cell loss, 1—small focal areas of neuronal cell loss, 2—columnar damage in the cortex or moderate to severe cell loss in the other regions, 3—cystic infarction and gliosis). The sum score from different regions was calculated for each animal resulting in a total maximum score of 24. Tissue atrophy was determined by measurement of intact areas in ipsi- and contra-lateral hemispheres at a distance of 400 μm using Image J software (NIH, USA). Volumes were calculated for the total hemisphere and cortex between +1 and −2.6 mm from bregma, for the striatum between +1 and −0.6 mm from bregma and for the hippocampus between −0.6 and −2.6 mm from bregma. Tissue loss was determined by comparison with contralateral volumes according to the following equation: 1– (volume ratio (left vs. right)) × 100.

For analysis of neuronal, oligodendrocyte and vessel densities cryostat sections taken at the level of +0.2 to +0.3 mm from bregma were stained for neuronal nuclei (NeuN), oligodendrocyte transcription factor 2 (Olig2) and cluster of differentiation 31 (CD31), respectively. Proliferative responses, neural precursor cells, oligodendrocyte maturation, myelination, astrogliosis, and microglia were evaluated by staining of Ki67, doublecortin (DCX), adenomatous polyposis coli, clone CC1 positive (referred as CC1), O4, myelin basic protein (MBP), glial fibrillary acidic protein (GFAP), and ionized calcium-binding adaptor protein-1 (Iba-1), respectively. Micro- and astroglia were further analyzed for co-expression of typical M1/M2 and A1/A2 markers, in the following co-staining: Iba-1/CD206 (M2), GFAP/complement C3 (A1), GFAP/pentraxin 3 (PTX3) (A2). Since CD86 (M1) staining with commonly suggested antibodies (i.e., abcam: ab119857, eBioscience: 14-0862-81) did not work together with Iba-1 staining in native tissue sections, microglia were identified by CD11b expression. To exclude contamination with CD11b^+^ peripheral leukocytes, CD45 was included in this co-staining. Detailed information on primary and secondary antibodies used, is provided in Supplementary Table 1. Immunohistochemistry was performed according to our previous studies (Drommelschmidt et al., 2016; Reinboth et al., 2016; Serdar et al., 2016; Herz et al., 2018b) with minor modifications. Briefly, tissue sections were thawed at 37°C for 15 min followed by fixation in 4% paraformaldehyde (PFA) for 5 min [NeuN, Olig2, CD31, Ki67, CC1, MBP, O4, GFAP (host: mouse) C3] or ice cold aceton/methanol [GFAP (host: rat), PTX3, CD45, CD11b, CD86, DCX] for 5 min. For Iba-1 staining, sections were incubated with 4% PFA overnight followed by antigen retrieval in sodium citrate buffer at 100°C for 20 min followed by incubation with 5% normal goat serum (NGS), 0.2% Tween 20 in phosphate buffered saline (PBS) for 30 min at room temperature. For CD45/CD11b/CD86 co-staining, tissue sections were incubated with 2% NGS, 1% bovine serum albumin (BSA) in 0.2% Tween in PBS for 30 min followed by anti-CD45 antibody incubation overnight at 4°C. For the other staining, unspecific antibody binding was blocked by incubation with 1% BSA, 0.3% cold fish skin gelatin (Sigma Aldrich, Germany), 0.2% Tween in PBS for 1 h at room temperature followed by primary antibody incubation overnight at 4°C. Antibody binding was visualized by incubation with appropriate anti-rat/mouse/rabbit/goat Alexa Flour 488, Alexa Flour 555 or Alexa Flour 647 conjugated secondary antibodies (all:1:500, Thermo Scientific, Germany) for 2 h at room temperature. For CD45/CD11b/CD86 co-staining, secondary antibody incubation (for CD45) was combined with fluorescein isothiocyanate (FITC)-conjugated anti-mouse CD11b and phycoerythrin (PE)-conjugated anti-mouse CD86 for 3 h. Nuclei were counterstained with 4′,6-Diamidin-2-phenylindol (DAPI, 100 ng/ml; Molecular Probes, USA).

Confocal imaging with the perfect focus system module (A1plus, Eclipse Ti, with NIS Elements AR software, Nikon, Germany) was performed to generate large scale images (stitching) of the complete striatum of NeuN-, Olig2-, CD31-, Ki67-, GFAP-, and Iba-1-stained tissue sections. Using the 20x objective z-stacks of 12 μm thickness (3 μm focal plane distance) were acquired in 7 × 6 overlapping regions (15% overlap). Images were converted into maximal intensity projections for automated software-based quantification using the NIS Elements AR software. Analysis was performed in entire striatum and the sub-ventricular zone (SVZ), which were demarcated on the basis of nuclei staining (DAPI) prior to measurement. Unbiased software based object detection was used to determine the number of NeuN^+^, Olig2^+^, Ki67^+^ cells, and CD31^+^ vessels. For assessment of oligodendrocyte maturation 2 defined non-overlapping regions of interest (ROIs) in the external capsule (each 135370 μm^2^) and 3 ROIs in the striatum (each 2,15,000 μm^2^) were analyzed to determine the number of Olig2/CC1, Olig2/O4 double, and Olig2 single positive cells. Myelination was quantified in the aforementioned ROIs by measurement of MBP-positive areas. Single object counting was not possible for Iba-1 and GFAP staining due to intensive local accumulation of microglia and glial scar formation by astrocytes in severely injured regions and animals (**Figure 4**). Therefore, positively stained areas and mean fluorescence intensities were quantified as a measure of cell density and activation, respectively (Pekny and Nilsson, 2005; Kozlowski and Weimer, 2012). To determine the expression level of typical A1/A2 markers, GFAP-positively-stained areas were automatically identified with the binary tool of the NIS Elements AR software followed by measurement of mean fluorescence intensities of C3 and PTX3 immunostaining on GFAP^+^ areas. Using the same approach, CD86 immunostaining was quantified on CD11b positive and CD45 negative/low areas to exclude confounding effects by peripheral immune cells. While CD86 immunostaining was predominantly found in regions of dense microglia accumulation/severe tissue injury, not allowing single cell analysis, CD206 was expressed on distinct cells mainly at the border of injured areas (**Figure 4B**). For quantification, CD206/Iba-1 double positive cells were counted manually. For quantification of A1/A2 and M1/M2 marker expression, 4 non-overlapping ROIs (each 390.600 μm^2^) were analyzed for each animal.

### mRNA Expression Analysis

For mRNA expression analyses in ipsi- and contralateral tissue parts, one 160 μm thick tissue section/animal were collected at the striatal level (0.5 mm to 0 mm from bregma). Total RNA was isolated with the RNeasy Micro Kit (Qiagen Germany) according to the manufactures recommendations. First strand complementary DNA was synthesized using 0.6 μg of total RNA and TaqMan reverse transcription reagents (Applied Biosystems/Thermo Fisher Scientific). Polymerase chain reaction (PCR) was performed in duplicates in 96 well-optical reaction plates for 40 cycles with each cycle at 94°C for 15 s and 60°C for 1 min using the StepOnePlus Real Time PCR system (Applied Biosystems/Thermo Fisher Scientific). PCR products were quantified using assay on demand primers and fluorogenic reporter oligonucleotide probes (Applied Biosystems/Thermo Fisher Scientific, Supplementary Table 2). CT values were normalized for the housekeeping gene beta-2-microglobulin [ΔCT = CT (target gene)-CT (beta-2-microglobulin)] and related to the mean of either sham-operated (Supplementary Figure 1) or vehicle-treated animals (**Figures 4**, **5**) using the ΔΔCT formula [ΔΔCT = ΔCT (sham/vehicle)-ΔCT (MSC-EV)]. Fold change values were calculated.

### Statistical Analysis

All results are expressed as box plots with individual data points including median values, the 25% and the 75% percentile. For statistical analysis, the GraphPad Prism 6.0 software package (GraphPad Software) was used. Data were tested for Gaussian distribution and analyzed either by ordinal 1-way ANOVA or by Kruskal-Wallis (non-parametric) with *post-hoc* Sidak's or Dunn's multiple comparison tests, respectively. When 2 groups were compared, unpaired, two-tailed Student *t-*test or Mann Whitney test (non-parametric) were applied. In all analyses, *p* < 0.05 was considered statistically significant. Exploratory pilot data (Supplementary Figure 1) are presented as scatter plots (immunohistochemistry) or box plots (mRNA expression). *P*-values derived from unpaired, two-tailed Student *t-*test (immunohistochemistry) or one sample *t*-test (mRNA expression) are depicted in the graphs.

## Results

### MSC-EVs Reduce HI-Induced Subacute Striatal Tissue Injury

**Figure 1:**
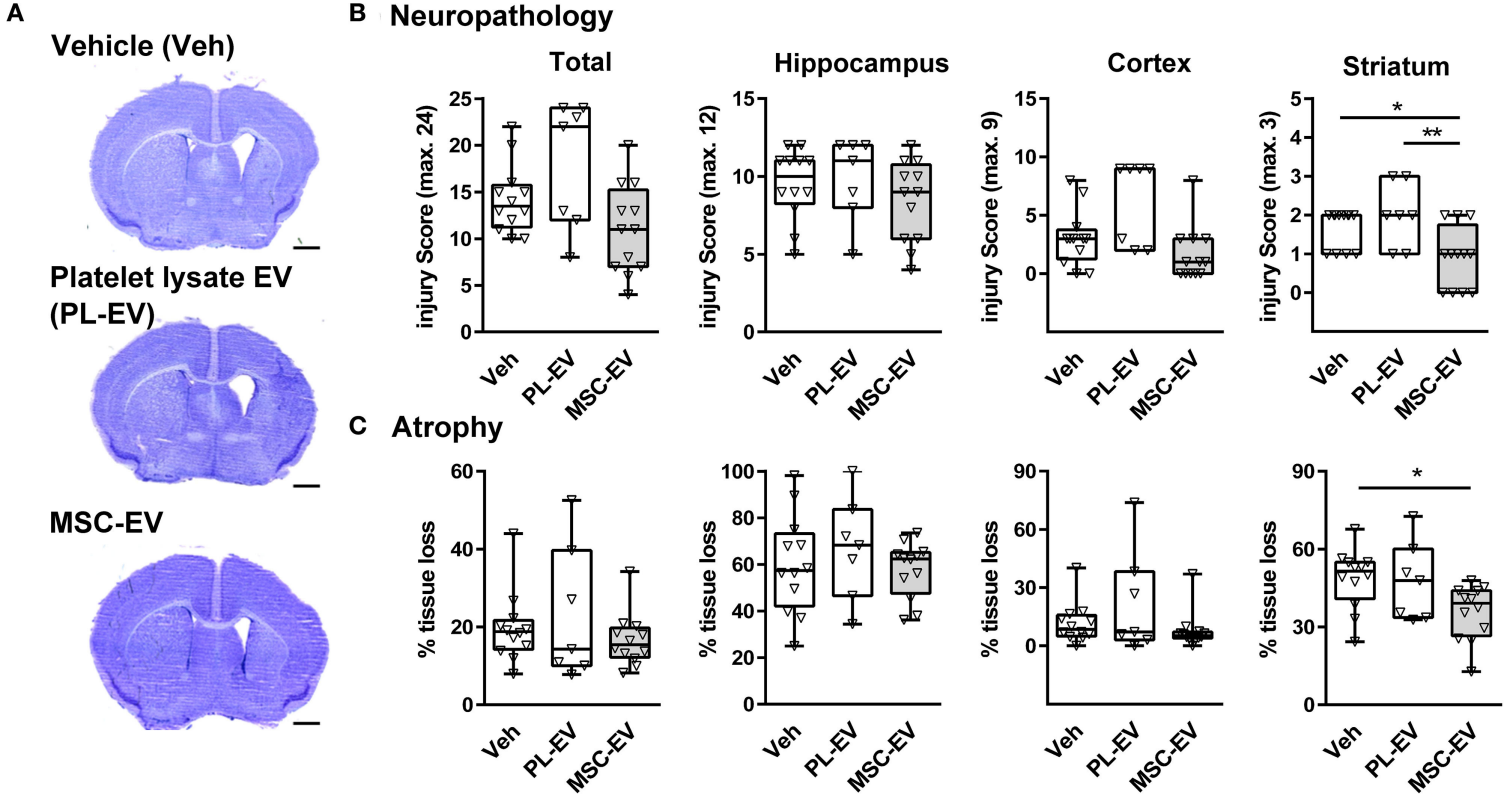
MSC-EVs reduce HI-induced striatal tissue injury. Histological brain injury was determined on cresyl violet stained 20 μm cryostat sections 7 days after HI, performed in 9-day-old C57BL/6 mice. Vehicle (0.9% NaCl, Veh), platelet-derived EVs (PL-EV), and MSC-EVs were administered i.p. 24, 72, and 120 h after HI. **(A)** Representative images of injured hemispheres for each experimental group are shown (scale bar: 1 mm). **(B)** Injury scores were assessed in different brain regions (cortex, hippocampus, and striatum) resulting in a sum score (total) quantified for each animal. **(C)** Brain atrophy was analyzed by measurement of intact areas on tissue sections at a distance of 400 μm between + 1 mm and – 2.6 mm from bregma. Volumes were calculated for total hemispheres and the indicated brain regions. Tissue loss was expressed as the percentage of volume reduction compared to intact contralateral volumes. *p < 0.05, **p < 0.01, *n* = 12 (Veh), *n* = 7 (PL-EV), *n* = 12 (MSC-EV).

To evaluate the impact of MSC-EVs on HI-induced brain injury we analyzed histopathological changes (Figures 1A,B) and brain atrophy (Figures 1A,C) in cresyl violet stained tissue sections (Figure 1A) 1 week after HI. In addition to vehicle control (Veh, 0.9% NaCl), we included EVs isolated from human platelet lysate (PL-EV), because our human MSCs were cultured in medium supplemented with platelet lysate, a rich source of EVs (Hemeda et al., 2014). While total neuropathological changes and atrophy were not modulated by MSC-EVs and PL-EVs, MSC-EVs but not PL-EVs significantly decreased pathological changes and tissue atrophy in the striatum (Figures 1B,C).

### MSC-EVs Increase Neuronal and Endothelial Cell Densities

**Figure 2:**
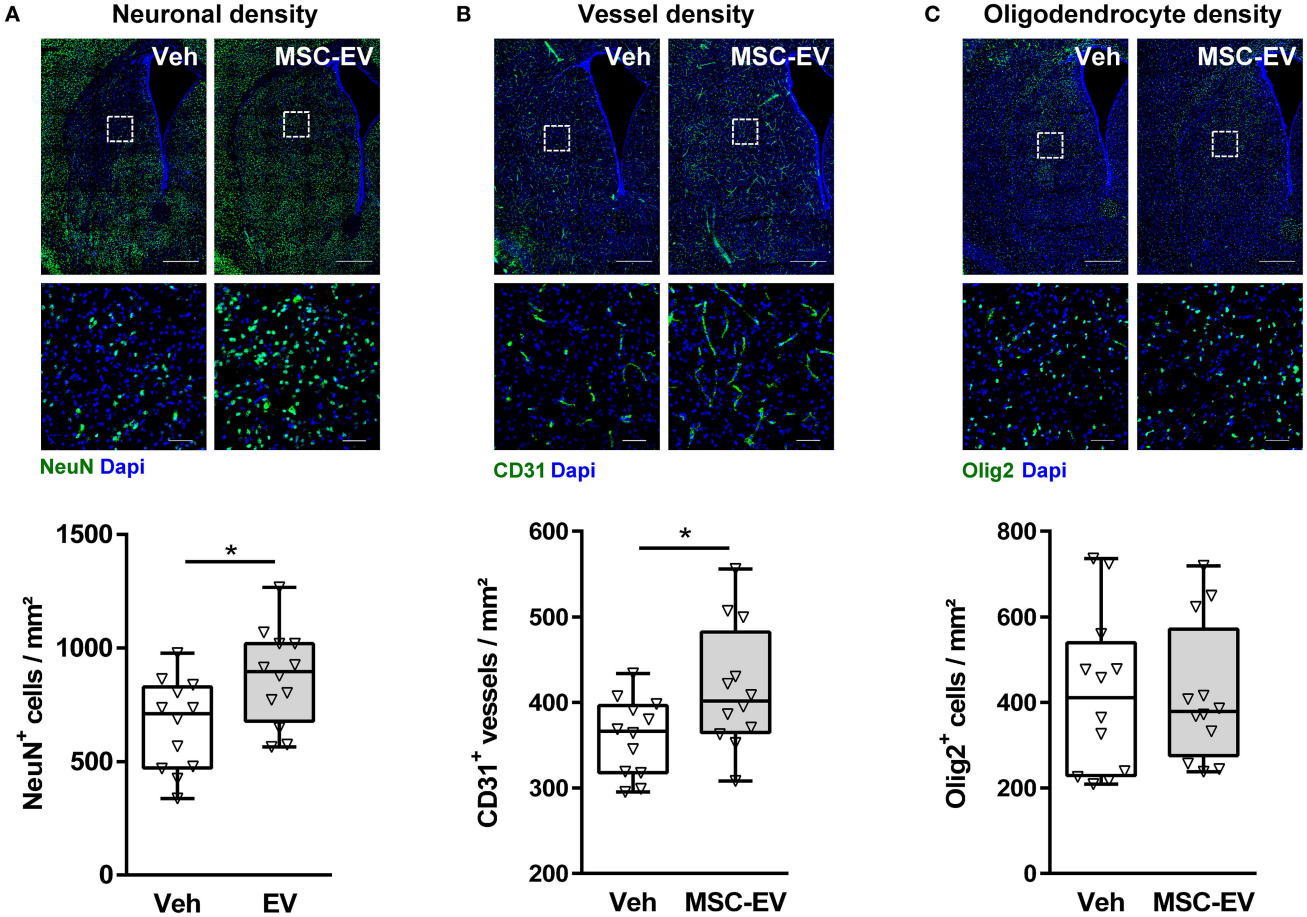
MSC-EVs increase neuronal and vessel densities 1 week after HI. Neuronal **(A)**, vessel **(B)**, and oligodendrocyte **(C)** densities were analyzed via immunohistochemistry for NeuN, CD31, and Olig2, respectively. Analysis was carried out in stitched large scale confocal images of the striatum obtained from brain sections of 16-day-old C57BL/6 mice that were exposed to HI on postnatal day 9. Vehicle (0.9% NaCl, Veh) and MSC-EVs were administered i.p. 24, 72, and 120 h after HI. White squares in large scale representative images (scale bar: 500 μm) indicate the location of high resolution images (scale bar: 50 μm) shown below. Cellular densities were quantified by unbiased automated software-based object detection. *p < 0.05, *n* = 12/group.

Neuropathological assessment and volumetric measurements in cresyl-violet stained sections provide a rough estimate of global HI-induced brain injury. However, cellular responses differ, e.g., while HI induces a significant reduction in neuronal and vessel densities; the number of oligodendrocytes increases (Supplementary Figure 1A; Reinboth et al., 2016). To get deeper insight, which cell types have been protected by MSC-EVs, we quantified neuronal (Figure 2A), vessel (Figure 2B), and oligodendrocyte (Figure 2C) densities in the striatum. Since potential confounding effects by PL-EVs in MSC-EV preparations could be excluded (Figure 1), we focused on the comparison between MSC-EV- and vehicle-treated animals. MSC-EVs lead to a significant increase in neuronal (Figure 2A) and vessel density (Figure 2B), while the amount of oligodendrocytes was not modulated (Figure 2C).

### MSC-EVs Enhance Cell Proliferation in the Neurogenic Sub-ventricular Zone (SVZ)

**Figure 3:**
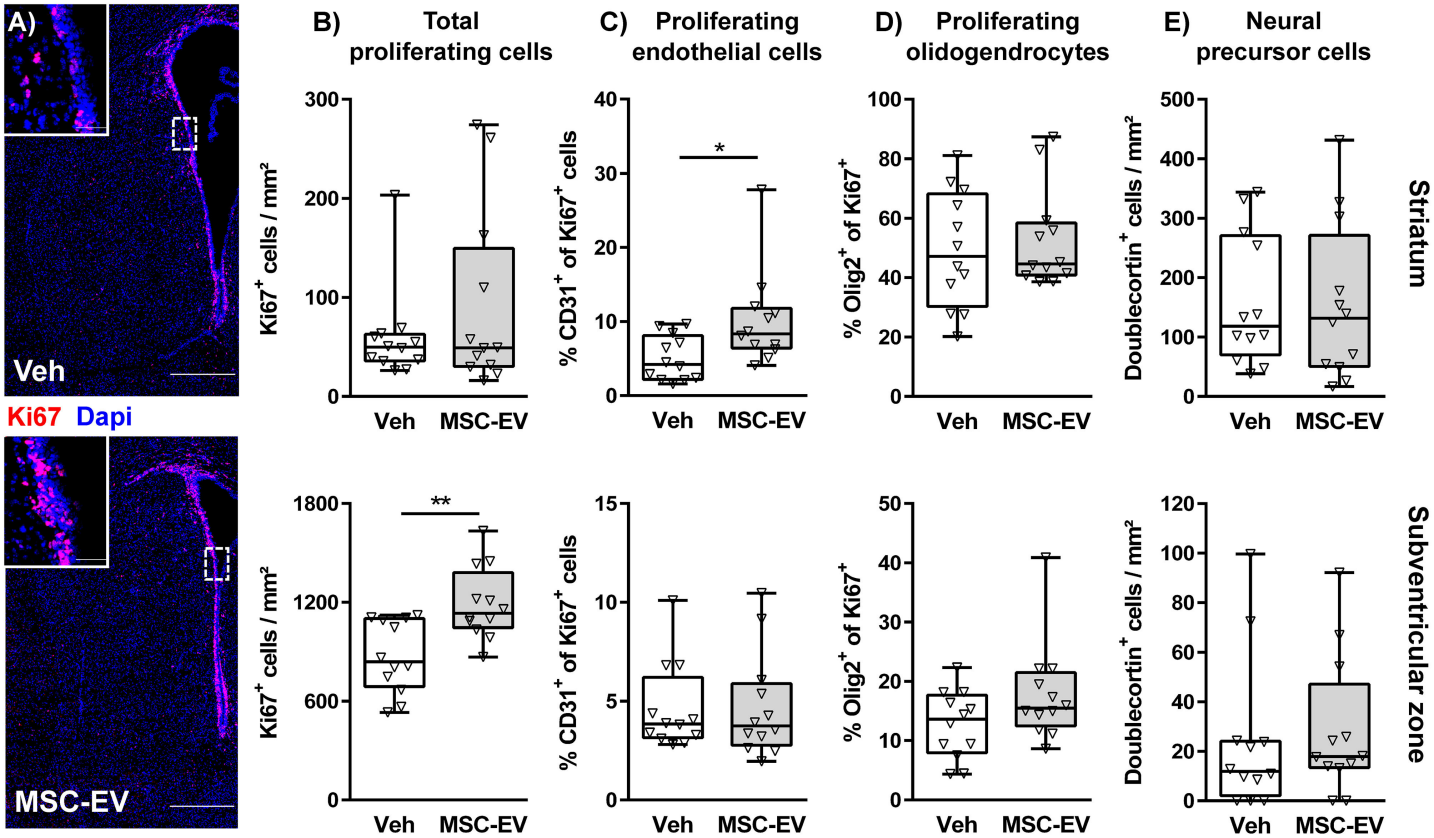
MSC-EVs increase regenerative proliferation. Cell proliferation was analyzed 7 days after HI in C57BL/6 mice exposed to HI on postnatal day 9. Vehicle (0.9% NaCl, Veh) and MSC-EVs were i.p. injected 24, 72, and 120 h after HI. The amount of proliferating cells was determined via immunohistochemistry for the proliferation marker Ki67 in the striatum and the subventricular zone **(A,B)**. The percentage of proliferating endothelial cells **(C)** and oligodendrocytes **(D)** from all proliferating cells was quantified in tissue sections co-labeled for the endothelial cell marker CD31 and the oligodendrocyte marker Olig2, respectively. No co-labeling could be detected in co-staining of the neural precursor cell marker doublecortin and Ki67. Therefore, the absolute amount of DCX positive cells was quantified **(E)**. *p < 0.05, **p < 0.01, *n* = 12/group, scale bar in **(A)**: 500 μm (inset: 50 μm).

According to the selective protection of the striatum, juxtaposed to the neurogenic niche of the SVZ, we next investigated neuroregenerative responses, differentiating between the striatum and the SVZ. Quantification of cellular proliferation via immunohistochemistry for the pan proliferation marker Ki67 demonstrated that HI induces an endogenous reparative response with an increased amount of proliferating cells in the striatum but not in the neurogenic SVZ (Supplementary Figure 1B). However, MSC-EV treatment lead to a significant increase of proliferating cells in the SVZ (Figures 3A,B). To identify proliferating cells we performed co-staining with the pan endothelial marker CD31 (Figure 3C) and the pan oligodendrocyte marker Olig2 (Figure 3D and Supplementary Figure 1B). To correct for the differences in overall Ki67^+^ cell density (Figures 3A,B and Supplementary Figure 1B), we quantified the percentage of CD31^+^ and Olig2^+^ cells of Ki67^+^ cells in the respective regions. Though total proliferating cells in striatum of HI-injured animals were increased compared to sham-animals, the percentage of CD31^+^ cells of all proliferating cells was reduced, coinciding with the observed decreased vessel density (Supplementary Figure 1A). Interestingly, increased vessel densities in the striatum of MSC-EV-treated animals (Figure 2B) were associated with a significantly increased frequency of proliferating CD31^+^ cells (Figure 3C). The proportion of oligodendrocytes in the total population of proliferating cells was not modulated, neither by HI nor by MSC-EV treatment (Figure 3D and Supplementary Figure 1B). To determine the contribution of neural precursor cell proliferation to the overall increase in cell proliferation in the striatum and the SVZ, we performed co-labeling with the neural precursor cell marker doublecortin (DCX). However, none of the DCX positive cells was labeled with Ki67. Furthermore, except of a slight increase of DCX^+^ cells in the SVZ following HI (Supplementary Figure 1B), the amount of DCX^+^ cells was not significantly modulated by MSC-EV-treatment (Figure 3E).

### MSC-EVs Decrease Astro- and Microgliosis and Modulate Inflammatory Cytokine and Neural Growth Factor Expression

**Figure 4:**
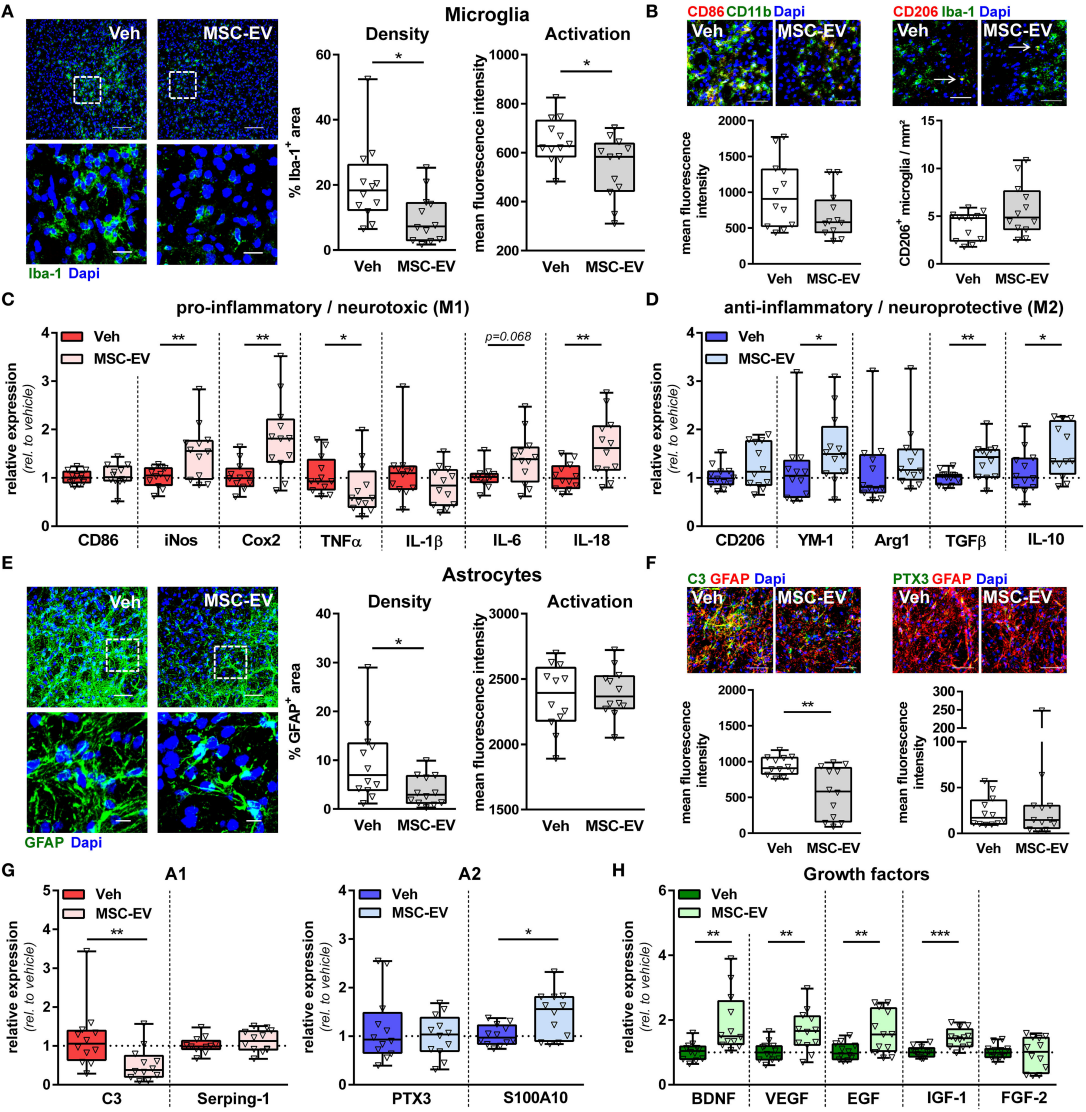
MSC-EVs reduce astro- and microglia activation and improve oligodendrocyte maturation. Nine-day-old C57BL/6 mice were exposed to HI followed by i.p. injection of MSC-EVs or vehicle (0.9% NaCl, Veh) 24, 72, and 120 h post-HI. Microglia density and activation was analyzed in stitched large scale confocal images of the striatum, obtained from brain sections stained for the microglia marker Iba-1 **(A)**. Low magnification images (scale bar: 100 μm) reveal maximal intensity projections of 12 μm z-stacks (plane distance 3 μm). To demonstrate morphology, high magnification images (scale bar: 20 μm) were acquired at a single plane in the white square, depicted in low magnification images. Iba-1 immunoreactivity, as a measure of microglia activation, was quantified by measurement of positively stained areas and fluorescence intensities in positively stained areas **(A)**. Expression of typical M1 (CD86) and M2 (CD206) markers was analyzed in CD11b (green)/CD86 (red) and Iba-1 (green)/CD206 (red) staining (**B**, scale bar: 50 μm). CD45-staining (not shown) was included in CD11b/CD86 co-staining to exclude confounding effects by peripheral immune cells, i.e., CD86 expression was only quantified on CD11b^+^ CD45^−^ areas. While CD86 was found predominantly in regions of dense microglia accumulation, CD206^+^ microglia were mainly found at the border region, which could be identified as discrete double positive cells (arrows, **B**). A broad set of pro-inflammatory M1-phenotype-associated **(C)** and anti-inflammatory M2-phenotype-associated **(D)** molecule expression was analyzed via real-time PCR in brain tissues (160 μm thickness) obtained from the striatal level (0.5–0 mm from bregma). Beta-2-microglobulin served as housekeeping gene and fold change values compared to vehicle-treated control animals were calculated. Astrocyte density and activation was analyzed in GFAP-stained brain tissue sections **(E)**. Analyses and acquisition of representative images was performed as described for microglia **(A)**. Expression of typical A1 (C3, green) and A2 (PTX3, green) markers was analyzed in co-staining with GFAP (red, **F**, scale bar: 50 μm). Immunohistochemistry results were confirmed by mRNA expression analysis as described for microglia including further typical A1 (Serping-1) and A2 (S100A10) markers **(G)** and essential neural growth factors **(H)**. *p < 0.05, **p < 0.01, ***p < 0.001, *n* = 12/group.

In addition to regenerative capacities, MSC-EVs have been suggested to reduce neuroinflammatory responses in different models of adult and perinatal brain injury (Drommelschmidt et al., 2016; Sisa et al., 2019; Wang et al., 2020; Xin et al., 2020). A major hallmark of HI-induced neuroinflammation is the activation of astrocytes and microglia, associated with modulation of their phenotype and alterations in the release of major effector molecules, i.e., cytokines and growth factors (Supplementary Figures 1C–I). Analysis of astroglia and microglia activation by immunohistochemistry for GFAP and Iba-1, respectively, demonstrated that HI induces an increase in micro- and astroglia densities accompanied by increased expression of Iba-1 and GFAP, as a measure of cellular activation (Pekny and Nilsson, 2005; Kozlowski and Weimer, 2012) (Supplementary Figures 1C,F). MSC-EV treatment significantly reduced the GFAP^+^ and Iba-1^+^ area and Iba-1 expression intensity (Figure 4A).

Microglia and astrocytes can acquire different activation states and exert many functions, contributing to both, brain damage and repair mechanisms (Ransohoff and Perry, 2009; Sofroniew, 2015; Liddelow and Barres, 2017). Being aware about the difficulty of terminology to describe the different activation states (Murray et al., 2014; Liddelow and Barres, 2017), we refer to the commonly used nomenclature of M1/M2 and A1/A2. M1/A1 cells are supposed to mediate pro-inflammatory/neurotoxic effects, while M2/A2 cells are supposed to reveal an anti-inflammatory and pro-regenerative phenotype (Ransohoff and Perry, 2009; Sofroniew, 2015; Liddelow and Barres, 2017). Immunohistochemistry analyses revealed that neonatal HI leads to an upregulation of the M1-cell surface marker CD86 on microglia cells, while no differences were observed for the amount CD206^+^ microglia compared to sham-operated animals (Supplementary Figure 1D). MSC-EV treatment did not modulate expression of both markers (Figure 4B). To quantify a broader set of typical M1/M2 molecules, we performed mRNA expression analyses in brain tissues obtained from the striatal level. These analyses demonstrated a strong upregulation of the pro-inflammatory cytokines tumor necrosis factor α (TNFα) and interleukin 1β (IL-1β) in HI-injured animals compared to sham controls (Supplementary Figure 1E). Of note, we also observed an upregulation of typical M2 markers, i.e., chitinase-like 3 protein (YM-1) and arginase-1 (Arg1) accompanied by reduced expression of typical M1 markers, i.e., inducible nitric oxide synthase (iNos) and cyclooxygenase 2 (Cox2) (Supplementary Figure 1E). Interestingly, HI-induced increase of TNFα expression and decreased iNos and Cox2 expression were reversed by MSC-EV therapy (Figure 4C). Furthermore, MSC-EV treatment lead to a significant elevation of the pro-inflammatory cytokine IL-18 and a further increase of the M2 marker YM-1 and the anti-inflammatory cytokine transforming growth factor β (TGFβ) (Figures 4C,D).

To characterize astrocytes more specifically, expression of the A1 protein complement C3 (C3) and the A2 protein pentraxin 3 (PTX3) were analyzed via immunohistochemistry. Neonatal HI lead to a strong but also moderate upregulation of C3 and PTX3, respectively (Supplementary Figure 1G). HI-induced upregulation of astrocytic C3 expression was significantly reduced in MSC-EV-treated animals, while PTX3 expression was not modulated (Figure 4F). To analyze further typical A1/A2 markers and prominent effector molecules of astrocytes (i.e., regenerative growth factors), mRNA expression analyses were performed. In line with results obtained from immunohistochemistry, both C3 and PTX3 mRNA expression were strongly upregulated, while the other two selected markers Serping 1 (A1) and S100 Calcium Binding Protein A10 [S100A10, (A2)] were not modulated by HI (Supplementary Figure 1H). Confirming results of astrocyte-specific protein expression analyses (Figure 4F), HI-induced increase in C3 expression was reversed by MSC-EV therapy and PTX3 expression was not altered (Figure 4G). Furthermore, the expression of the A2 marker S110A10 was significantly upregulated in MSC-EV-treated animals. As expected, neonatal HI lead to a significant reduction of important growth factors like brain derived growth factor (BDNF), vascular endothelial growth factor (VEGF) and epidermal growth factor (EGF) (Supplementary Figure 1I). Importantly, MSC-EV therapy significantly enhanced mRNA expression of these molecules including insulin like growth factor (IGF-1) (Figure 4H).

### MSC-EVs Promote Oligodendrocyte Differentiation and Myelination

**Figure 5:**
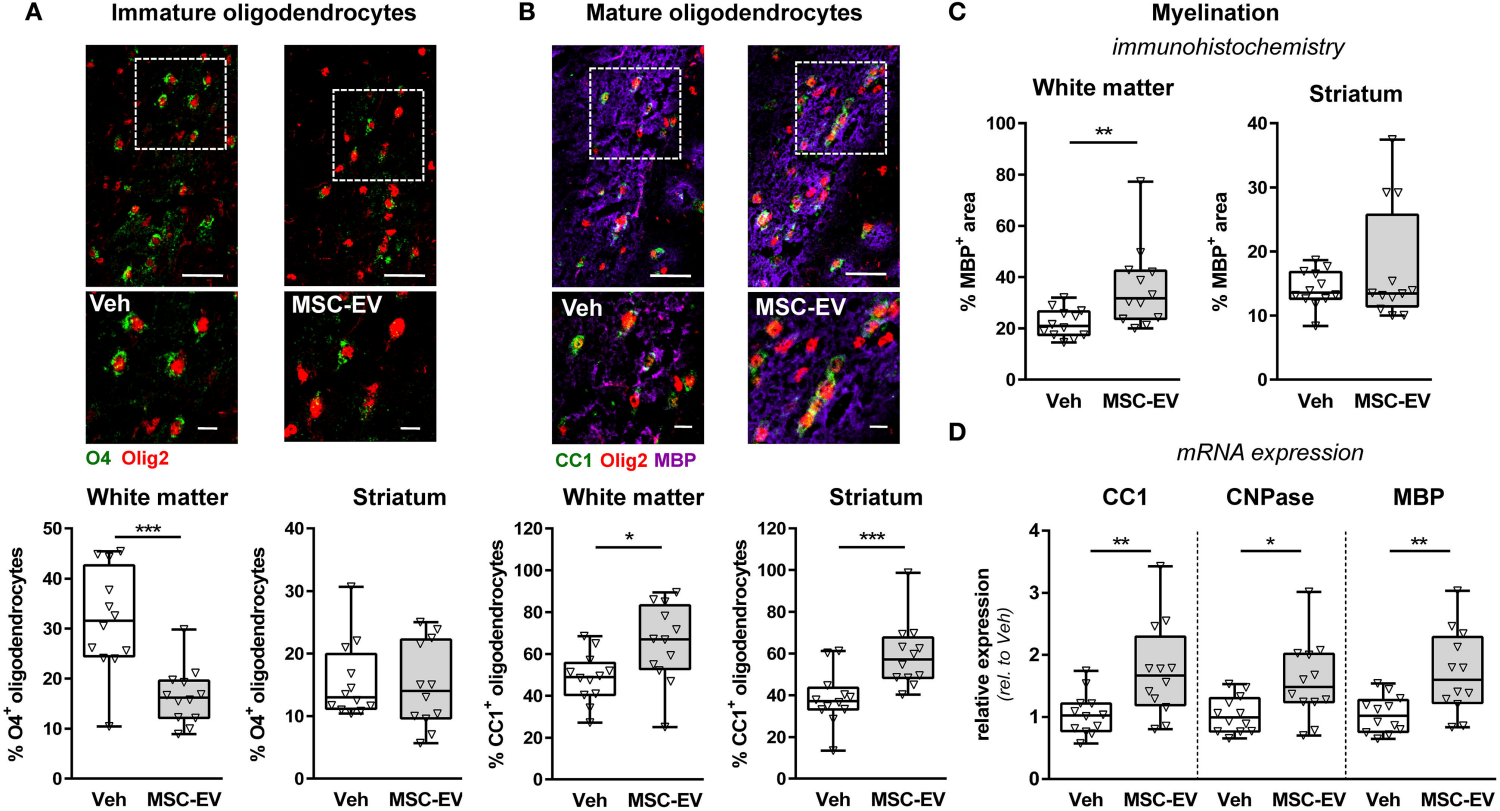
MSC-EVs improve oligodendrocyte maturation and myelination. Oligodendrocyte maturation and myelination was investigated in the white matter (external capsule) and the striatum via immunohistochemistry in co-staining for the pan-oligodendrocyte marker Olig2 (red, **A,B**) and either O4, labeling immature oligodendrocyte precursor cells (green, **A**) or CC1, labeling mature oligodendrocytes (green, **B**). Myelination was assessed by co-staining against MBP (violet, **B,C**). Representative images are obtained from the external capsule. Low magnification images (scale bar: 40 μm) reveal maximal intensity projections of 10 μm z-stacks (plane distance 2 μm). To demonstrate morphology, high magnification images (scale bar: 10 μm) were acquired at a single plane in the white square, depicted in low magnification images. The percentage of double positive cells from total oligodendrocytes was quantified **(A,B)**. Myelination was quantified by measurement of MBP^+^ areas **(C)**. Immunohistochemistry analyses were confirmed by mRNA expression analyses of CC1, CNPase and MBP in brain tissues (160 μm thickness) obtained from the striatal level (0.5 mm to 0 mm from bregma). Beta-2-microglobulin served as housekeeping gene and fold change values compared to vehicle-treated control animals were calculated **(D)** *p < 0.05, **p < 0.01, ***p < 0.001, *n* = 12/group.

Disruption of developmental processes by HI plays an important role in neonatal brain injury. A major contributor to neurodevelopmental outcome is myelination. Besides oligodendrocyte proliferation, oligodendrocyte maturation is essential for brain development and function. Therefore, we determined the number of immature O4^+^ (Figure 5A and Supplementary Figure 1J) and mature CC1^+^ (Figure 5B and Supplementary Figure 1K) oligodendrocytes in the striatum and the adjacent white matter in the external capsule. Myelination was evaluated via immunohistochemistry for MBP (Figure 5C and Supplementary Figure 1L). In line with our previous indications for an impaired oligodendrocyte maturation and disturbed myelination following neonatal HI (Reinboth et al., 2016), we detected a significantly reduced proportion of mature oligodendrocytes and reduced myelination in the white matter coinciding with a significant increase in immature oligodendrocytes (Supplementary Figures 1J,L). Of note, MSC-EVs significantly reduced the amount of immature O4^+^ oligodendrocytes in the white matter (Figure 5A), which was accompanied by a significant increase of differentiated CC1^+^ cells (Figure 5B) and enhanced MBP expression (Figure 5C). Results obtained from immunohistochemistry analysis were confirmed by mRNA expression analysis for CC1, MBP and 2′,3′-cyclic-nucleotide 3′-phosphodiesterase (CNPase) in brain tissues derived from the striatal level, revealing a strong reduction by neonatal HI compared to uninjured sham-operated animals for all investigated analytes (Supplementary Figure 1L). Importantly, these HI-induced deficits were significantly improved after MSC-EV therapy (Figure 5D).

## Discussion

The neuroprotective potential of MSC-EVs has been shown in different models of adult and neonatal brain injury (Doeppner et al., 2015; Drommelschmidt et al., 2016; Sisa et al., 2019; Wang et al., 2020). However, for neonatal HI, only limited data are available with partially discrepant study results (e.g., microglia activation; Ophelders et al., 2016; Sisa et al., 2019; Xin et al., 2020). Furthermore, common to the majority of studies was a major focus on the early phase after injury. In the present study, we investigated the impact of MSC-EVs in the subacute disease phase, when inflammatory processes, delayed cell death but also endogenous regenerative processes are overlapping (Ferriero, 2004). We demonstrate that MSC-EVs protect from HI-induced striatal tissue loss, associated with an increased neuronal and vessel density and a significant increase of proliferating cells in the neurogenic sub-ventricular zone juxtaposed to the striatum. Furthermore, MSC-EV treatment reduced astroglia and microglia activation and increased endothelial cell proliferation and oligodendrocyte maturation.

Regional differences in vulnerability to HI-induced brain injury and therapeutic interventions are well-known (Northington et al., 2011; Reinboth et al., 2016; Sisa et al., 2019). With regard to therapeutic effects of MSC-EVs, Sisa et al. observed significant protection from apoptosis and volume loss in the cortex, external capsule and thalamus (Sisa et al., 2019). We also observed regional differences, with major protection from brain injury in the striatum, but not in the cortex. Discrepancy in protected brain regions might not only be explained by differences in administration route (intranasal vs. intraperitoneal), but also by different time points of intervention and analysis (intervention: 0 vs. 24, 72, and 120 h; analysis: 2 vs. 7 days). Indeed, our previous study provided evidence for a spatial–temporal regulation of pathophysiological processes during the evolution of HI and for different therapeutic time windows in individual brain structures (Reinboth et al., 2016). In the present study, particularly the striatum was protected, which might be explained by the close proximity to the neurogenic sub-ventricular zone, where MSC-EVs promoted regenerative proliferation. Nevertheless, further in depth analysis of other brain structures, e.g., the neurogenic sub-granular zone at the level of the hippocampus may fully uncover the neuro-regenerative potential of MSC-EV therapy. Furthermore, whether protection in the striatum may lead to subsequent protection from thalamic injury and/or of striatal-thalamic projections needs to be analyzed in future studies.

Regenerative processes in the sub-acute disease phase include neurogenesis and angiogenesis. Similarly as in adult ischemic brain injury (Doeppner et al., 2015), we observed an increased proliferative response in neurogenic niches of the brain after MSC-EV-therapy. Increased proliferation in the sub-ventricular zone juxtaposed to the striatum may explain higher neuronal and vessel densities detected in the striatum. However, with our co-labeling strategies for oligodendrocytes, endothelial cells and neural precursor cells we could only assign 15% of total proliferating cells to a specific cell type. The amount of doublecortin positive cells, a major neural precursor cell marker, was not modulated by MSC-EV treatment. These results indicate that increased neuronal cell numbers in the striatum may be rather attributed to increased neuronal survival than to generation of new neurons. Further work investigating a broader set of markers for neuronal precursor cell types is needed to fully elucidate the impact of MSC-EVs on neurogenesis in neonatal HI. Nevertheless, in the present study we also show that MSC-EVs induce striatal angiogenesis, an important hallmark of post-ischemic neuroregeneration and neuronal network formation during brain development (Coelho-Santos and Shih, 2020; Hatakeyama et al., 2020). Thus, increased striatal angiogenesis after MSC-EV treatment might contribute to overall increased tissue recovery.

In addition to angiogenesis, myelination is key to the functional activity of axons, allowing them to connect to neurons and strengthen circuitry throughout nervous system development. Clinical and our preclinical studies revealed a strong association between oligodendrocyte maturation and long-term motor-cognitive neurodevelopment in preterm birth-related brain injury (Counsell et al., 2008; Drommelschmidt et al., 2016; Serdar et al., 2016). In the context of HI at term-equivalent age, we have previously shown that HI induces oligodendrocyte proliferation, which however fails to improve myelination (Reinboth et al., 2016). This is confirmed by the present results, demonstrating an overall increase of oligodendrocytes, most likely due to enhanced proliferation. However, this endogenous regenerative response is limited, as these newly arising oligodendrocytes do not differentiate into mature oligodendrocytes to promote myelination. Importantly, MSC-EVs significantly improve oligodendrocyte maturation and myelination. Since the amount of total oligodendrocytes was not altered by MSC-EVs, effects on oligodendrocyte cell death and/or proliferation seem unlikely. MSC-EVs may rather modulate the maturity level of oligodendrocytes, promoting oligodendrocyte differentiation. This is reflected by a reduction of immature oligodendrocytes and a simultaneous increase of differentiated cells and an increased expression of typical myelin markers (e.g., MBP, CNPase). These data suggest that MSC-EVs may overcome HI-induced disturbance of neurodevelopmental processes, i.e., oligodendrocyte maturation and myelination, which is supported by the study of Sisa et al. demonstrating protective effects by MSC-EVs in the white matter (i.e., external capsule) in the early post-hypoxic disease phase.

In line with Sisa et al. and own previous work in inflammation-induced preterm-related brain injury (Drommelschmidt et al., 2016; Sisa et al., 2019), astro- and microgliosis were reduced following MSC-EV treatment. While the former studies focused on the acute phase after the injurious stimulus, we here demonstrate that also secondary inflammatory processes are modulated by MSC-EVs, thereby probably facilitating an environment for enhanced regeneration and thus improved long-term recovery (Giebel and Hermann, 2019). Nevertheless, Iba-1 and GFAP immunoreactivity provide only limited information about astro- and microglia phenotype and function. Recent work indicates that MSC-EVs modulate the ratio of typical pro-inflammatory/neurotoxic M1 and anti-inflammatory/neuroprotective M2 markers (Xin et al., 2020). Here, we demonstrate that HI-related alterations of typical M1/M2 markers was counter-regulated by MSC-EV therapy. For instance, HI-induced upregulation of the pro-inflammatory cytokine TNFα was significantly reduced after MSC-EV treatment, while expression of the anti-inflammatory cytokine TGFβ and the M2 marker YM-1 was significantly increased. These results suggest that MSC-EVs modulate microglia to promote anti-inflammatory and protective effects. However, in line with previous reports (Hellstrom Erkenstam et al., 2016), this strict M1/M2 classification concept based on single molecule expression might oversimplify the complexity of cellular responses *in vivo*. Indeed, in the present study, we detected a reduction of the typical M1 molecules iNos and Cox2, which were associated with increased neuroinflammatory and degenerative responses, particularly in the acute phase after ischemic brain injury (del Zoppo et al., 2000; Willmot et al., 2005). Nevertheless, our present results are in line with previous work demonstrating a trend to reduced expression of iNos and Cox2 at delayed time points after perinatal brain injury (i.e., 5–7 days after injury; Hellstrom Erkenstam et al., 2016; Chhor et al., 2017). Considering the important function of both enzymes and their products in physiological brain development (Hickey et al., 2007; Angelis et al., 2020), HI-induced reductions of iNos and Cox2 1 week after injury, may lead to a delay of neurodevelopmental processes. This is supported by our observations that tissue-protective effects of an MSC-EV therapy were associated with a significantly increased expression of iNos and Cox2.

Analysis of astrocyte responses revealed similar difficulties for a strict phenotype classification. HI induced a significant increase of both, A1-associated C3 and A2-assoicated PTX3, expression. While MSC-EV treatment significantly reduced C3 expression, PTX3 was not modulated. However, the regenerative capacity of MSC-EVs was demonstrated by an elevated expression of major astrocytic effector molecules, i.e., neural growth factors. Interestingly, increased BDNF expression after MSC-EV therapy coincided with an upregulation of the pro-inflammatory cytokine IL-18. This might be explained by the double-edged function of IL-18, which does not only promote neurodegeneration (Felderhoff-Mueser et al., 2005a,b), but also enhances BDNF production and neuronal survival upon *in vitro* hypoxic-ischemic injury (Zhou et al., 2014). In addition to BDNF, growth factors involved in angiogenesis, i.e., VEGF and EGF were significantly upregulated after MSC-EV therapy. These findings provide a plausible link to the observed increase in vascular density and endothelial proliferation after MSC-EV treatment.

The present study suggests that MSC-EVs modulate micro- and astroglia phenotypes, facilitating an anti-inflammatory and reparative/regenerative tissue environment for protection from HI-induced secondary brain injury and associated neurodevelopmental complications. Nevertheless, data need to be interpreted with caution, since mRNA expression was analyzed in total tissue lysates, not allowing conclusions about the cellular source of cytokines and growth factors. Furthermore, previous work in adult and neonatal brain injury demonstrated co-expression of different category markers (Vogel et al., 2013; Hellstrom Erkenstam et al., 2016), which cannot be determined in the present study. Another issue, which warrants further investigation, is to distinguish cause and consequence, i.e., to identify direct cellular targets of MSC-EVs in neonatal HI. Considering that TNFα released from activated microglia is one of the strongest A1 inducers (Liddelow et al., 2017), our observations of a simultaneous downregulation of TNFα and C3 indicate that MSC-EVs modulated microglia with a consequent modulation of astrocyte phenotype and function. In contrast, MSC-EVs may directly affect both, micro- and astroglia, involving similar molecular mechanisms, e.g., through miRNAs targeting astrocytes (Xin et al., 2013, 2017) and microglia (Xin et al., 2020). To differentiate between direct in indirect effects of MSC-EVs, appropriate *in vitro* models with purified cells, but also *in vivo* tracking analyses of systemically administered EVs will be indispensable. So far, the majority of studies, addressing this issue, applied EVs intravenously with most frequent accumulation in the liver, lung and spleen (Yi et al., 2020). However, bio-distribution depends on many different factors, including the kind of injury and the cellular source of EVs (Yi et al., 2020). With regard to intraperitoneal injection of MSC-EVs, as applied in the present study, data about bio-distribution are sparse. In a model of pancreatic cancer, it was demonstrated that MSC-EVs accumulated in the pancreas, liver, spleen and lung (Mendt et al., 2018). Though peripheral effects of EVs seem likely, HI-induced modulation of the blood brain barrier may facilitate increased accumulation of systemically administered MSC-EVs in the injured brain. Nevertheless, considering limitations of currently available labeling techniques due to potential modulation of EV function and labeling artifacts (Thery et al., 2018), additional work is needed, to identify direct cellular targets of MSC-EVs.

Taken together, the present results suggest that MSC-EVs increase neuronal survival and/or neurogenesis through modulation of micro- and astroglial cell responses and promotion of neurodevelopmental processes (i.e., myelination). We partially confirmed observations made in other models of brain injury, which may help to identify common cellular targets in the CNS (e.g., glial cells, endothelial cells). Nevertheless, comparisons between different studies are limited not only due to different pathologies and developmental stages, but also due to the well-known EV heterogeneity. Major contributors to MSC-EV heterogeneity are differences in MSC donors, MSC culture conditions and EV preparation (Lener et al., 2015; Borger et al., 2017; Witwer et al., 2019; Wang et al., 2020). In our previous study, we observed different therapeutic activities between MSC-EV preparations, although they were prepared with the same protocol (Wang et al., 2020). Since we administered MSC-EVs prepared from one single donor, our study results need to be confirmed with other EV preparations in future studies. In addition to donor differences, methods of MSC cultivation for EV preparation differ between laboratories, e.g., media supplements (Witwer et al., 2019). We used human platelet lysate (PL) as substitute for fetal calf serum. Since PL is a rich source of EVs (Hemeda et al., 2014), which co-purifies with MSC-EVs, PL-EVs may contribute to or confound therapeutic effects of MSC-EVs. While this control was not included in previous studies, our present results demonstrate that PL-EVs do not mediate therapeutic effects, at least in this specific experimental setting. Therefore, observed protective and supportive effects of MSC-EVs in our study can be attributed to EVs derived from MSCs.

Our study provides important new information to the field, because we have shown that MSC-EV therapy can be applied within a delayed therapeutic window and therefore, in principle, allows combination with the standard clinical care HT. This is particularly important since HT is less effective in improvement of white matter injury compared to MSCs (Herz et al., 2018b). Therefore, MSC-EVs combined with HT may not only overcome risks associated with cell therapy but also limitations of HT, which needs to be proven in future studies.

## Data Availability Statement

The original contributions presented in the study are included in the article/Supplementary Materials, further inquiries can be directed to the corresponding author/s.

## Ethics Statement

This animal study was reviewed and approved by State Agency for Nature, Environment and Consumer Protection North Rhine-Westphalia.

## Author Contributions

JH, BG, and IB conceptualized the study and designed the project. NK, CK, and JH performed experiments, analyzed, and interpreted data. YM, VB, UF-M, IB, and BG provided critical comments and suggestions during the drafting of the manuscript. JH wrote the manuscript. All authors contributed to the article and approved the submitted version.

## Conflict of Interest

The authors declare that the research was conducted in the absence of any commercial or financial relationships that could be construed as a potential conflict of interest. However, BG is scientific board member of Evox Therapeutics and Innovex Therapeutics SL.
