# Supplementary material for: Mesenchymal Stromal Cell-Derived Extracellular Vesicles Reduce Neuroinflammation, Promote Neural Cell Proliferation and Improve Oligodendrocyte Maturation in Neonatal Hypoxic-Ischemic Brain Injury
Source: Front Cell Neurosci. 2020 Dec 10;14:601176. doi: 10.3389/fncel.2020.601176 (PMC7758466; doi:10.3389/fncel.2020.601176)
Supplement: Supplementary file 1 [file Data_Sheet_1.pdf]

Supplemental Material

Mesenchymal stromal cell-derived extracellular vesicles reduce neuroinflammation, promote neural cell proliferation and improve oligodendrocyte maturation in neonatal hypoxic-ischemic brain injury

Nicole Kaminski[1]*, Christian Köster[1]*, Yanis Mouloud[2], Verena Börger[2], Ursula Felderhoff-Müser[1], Ivo Bendix[1], Bernd Giebel[2], Josephine Herz[1#]

[1] *Department of Pediatrics I, Neonatology & Experimental Perinatal Neurosciences, University Hospital Essen, University Duisburg-Essen, Essen, Germany*

[2] *Institute for Transfusion Medicine, University Hospital Essen, University Duisburg-Essen, Essen, Germany*

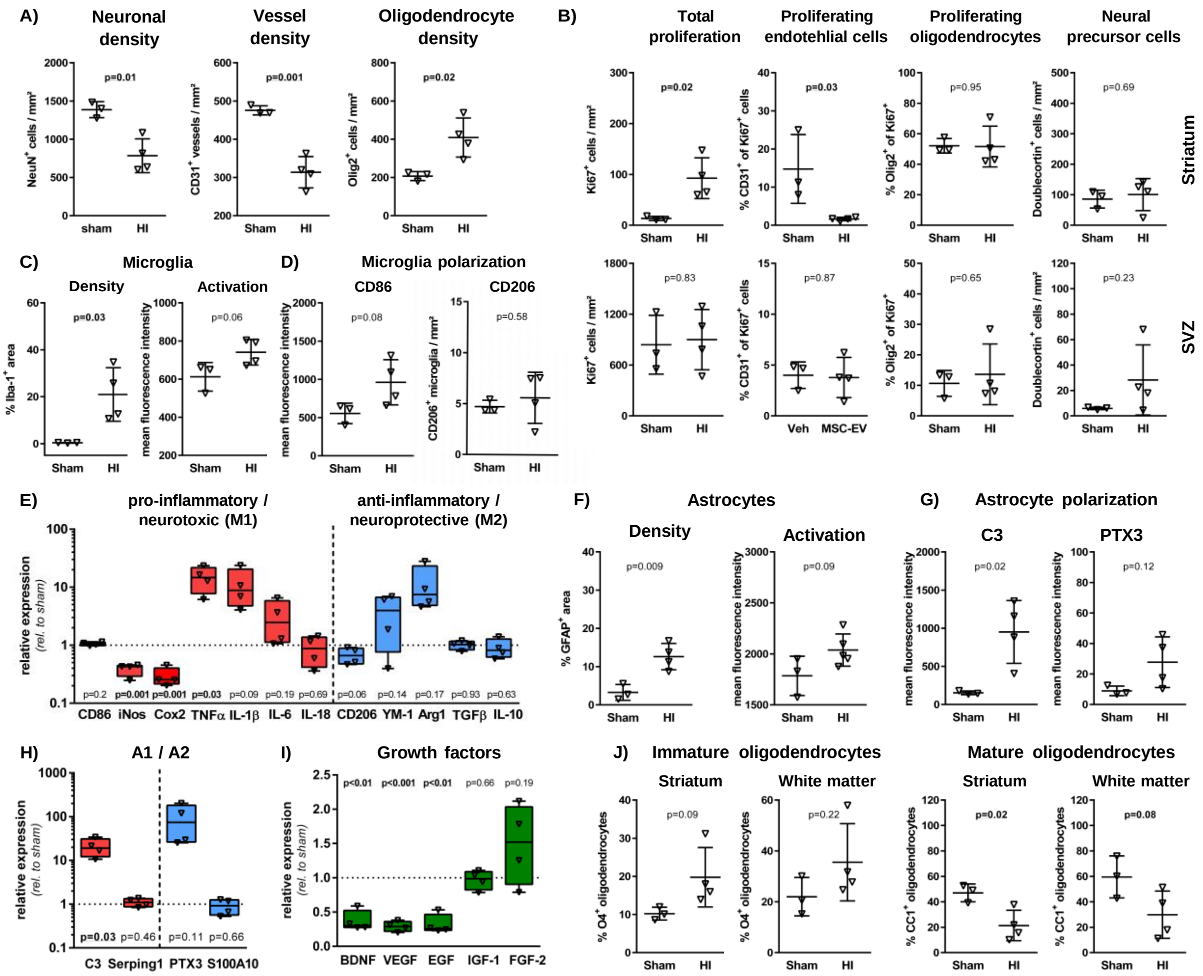

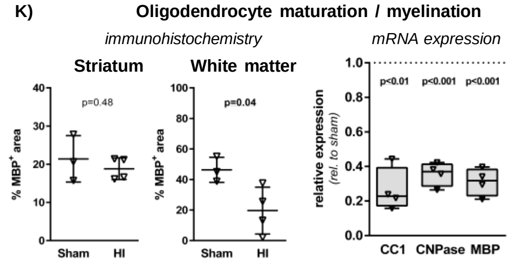

Supplemental Figure S1: Neuronal, endothelial and glial responses to neonatal hypoxia-ischemia. Nine-day-old C57BL/6 mice were exposed to hypoxia-ischemia (HI) or sham operation. Analysis was performed 7 days after HI. Neuronal, endothelial and glial cell responses were characterized by immunohistohistochemistry and mRNA expression analysis. **(A)** Neuronal vessel and oligodendrocyte densities were analyzed via immunohistochemistry for NeuN, CD31 and Olig2, respectively. Analysis was carried out in stitched large scale confocal images of the striatum. Cellular densities were quantified by unbiased automated software-based object detection. **(B)** Cell proliferation was analyzed in the striatum and the sub-ventricular zone in brain tissue sections stained for the proliferation marker Ki67. The percentage of proliferating endothelial cells and oligodendrocytes from all proliferating cells was quantified in tissue sections co-labelled for the endothelial cell marker CD31 and the oligodendrocyte marker Olig2, respectively. No co-labelling could be detected in co-staining of the neural precursor cell marker doublecortin (DCX) with Ki67. Therefore, the absolute amount of DCX positive cells was quantified. **(C)** Microglia density and activation was analyzed in stitched large scale confocal images of the striatum, obtained from brain sections stained for the microglia marker Iba-1. Iba-1 immunoreactivity was quantified by measurement of positively stained areas and fluorescence intensities in positively stained areas, as a measure of microglia activation **(D)**. Expression of typical M1 (CD86) and M2 (CD206) markers were analyzed in CD11b/CD86 and Iba-1/CD206 co-staining. CD45-staining was included in CD11b/CD86 co-staining to exclude confounding effects by peripheral immune cells, i.e. only CD11b+CD45- areas were used for quantification of CD86 expression. **(E)** A broad set of pro-inflammatory M1-phenotype-associated and anti-inflammatory M2-phenotype-associated molecule expression was analyzed via real-time PCR in brain tissues (160 µm thickness) obtained from the striatal level (0.5 mm to 0 mm

from bregma). Beta-2-microglobulin served as housekeeping gene and fold change values compared to sham-treated control animals were calculated. **(F)** Astrocyte density and activation was analyzed in GFAP-stained brain tissue sections. Analyses were performed as described for microglia (C). **(G)** Expression of typical A1 (C3) and A2 (PTX3) markers were analyzed in co-staining with GFAP. **(H)** Immunohistochemistry results were confirmed by mRNA expression analysis performed as described for microglia including further typical A1 (Serping-1) and A2 (S100A10) markers. **(I)** Aforementioned mRNA expression analyses also included quantification of essential neural growth factors. **(J-L)** Oligodendrocyte maturation and myelination was investigated in the white matter (external capsule) and the striatum via immunohistochemistry in co-staining for the pan-oligodendrocyte marker Olig2 with either O4 labelling immature oligodendrocytes **(J)** or CC1, labelling mature oligodendrocytes **(K)**. Myelination was assessed by co-staining against MBP (**L**, left). The percentage of double positive cells from total oligodendrocytes was quantified **(J, K)**. Myelination was quantified by measurement of MBP^+ areas (**L**, left). Immunohistochemistry analyses were confirmed by mRNA expression analysis of CC1, CNPase and MBP in brain tissues (160 µm thickness) obtained from the striatal level (0.5 mm to 0 mm from bregma). Beta-2-microglobulin served as housekeeping gene and fold change values compared to vehicle-treated control animals were calculated **(L)**. *p<0.05, **p<0.01, ***p<0.001, n=12/group

Supplemental Tables

Supplemental Table S1: Antibodies used for immunohistochemistry

antigen	dilution	reactivity	host	supplier	catalog number
NeuN	1:100	mouse	rabbit	Millipore	ABN78
CD31	1:200	mouse	rat	BD Biosciences	550274
Olig2	1:100	mouse	rabbit	Millipore	AB9610
Ki67	1:250	mouse/rat	rabbit	Abcam	ab66155
Ki67*	1:100	mouse	rat	Thermo	14-5698-82
Doublecortin	1:50	mouse	mouse	Santa Cruz	sc-271390
APC-CC1	1:100	mouse/rat	mouse	Calbiochem	OP80
MBP	1:150	mouse/rat	rat	Abcam	ab7349
O4	1:500	mouse/rat	mouse	Neuromics	MO15002
Iba-1	1:500	mouse/rat	rabbit	Wako	019-19741
GFAP	1:500	mouse	rat	Invitrogen	13-0300
GFAP**	1:500	mouse	mouse	Convance	SMI-22
CD86 PE	1:20	mouse	rat	BD Pharmingen	561963
CD11b FITC	1:100	mouse	rat	BD Pharmingen	557396
CD45	1:100	mouse	rat	BD Pharmingen	550539
CD206	1:500	mouse	goat	R&D Systems	AF2535
C3	1:50	mouse	rat	Abcam	ab11862
PTX	1:200	mouse	rabbit	Enzo	ALX-210-365-C050

**for co-labelling with anti-Olig2 and anti-NeuN*

***for co-labelling with anti-C3*

Supplemental Table S2: TaqMan Assays used for mRNA expression analyses

Gene	Abbreviation	Assay ID
pentraxin-3	PTX3	Mm00477268_m1
complement C3	C3	Mm01232779_m1
s100 calcium binding protien A10	S100A10	Mm00501458_g1
serping-1	Serping1	Mm00437835_m1
fibroblast growth factor – 2	FGF2	Mm01285715_m1
cluster of differentiation 86	CD86	Mm00444543_m1
cluster of differentiaion 206	CD206	Mm01329362_m1
vascular endothelial growth factor a	VEGF	Mm00437306_m1
2′, 3′-cyclic nucleotide 3′-phosphodiesterase	CNPase	Mm01306640_m1
cyclooxygenase-2	Cox2	Mm03294838_g1
adenomatous polyposis coli	CC1	Mm00545877_m1
brain-derived neurotrophic factor	BDNF	Mm01334043_m1
interleukin-1beta	IL-1β	Mm00434228_m1
epidermal growth factor	EGF	Mm00438696_m1
interleukin-6	IL-6	Mm00446190_m1
chitinase-like protein 3	YM1	Mm00657889_mH
nitric oxide synthase 2	iNos	Mm00440502_m1
arginase	Arg1	Mm00475988_m1
insulin-like growth factor I	IGF1	Mm00439560_m1
transforming growth factor beta-1	TGFβ	Mm01178820_m1
interleukin-18	IL-18	Mm00434226_m1
myelin basic protein	MBP	Mm01266402_m1
interleukin-10	IL-10	Mm01288386_m1
tumor necrosis factor alpha	TNFα	Mm00443258_m1
beta-2-microglobulin	B-2m	Mm00437762
